# Comparative estimation of nitrogen in urea and its derivative products using TKN, CHNS and hand-held refractometer

**DOI:** 10.1038/s41598-022-15736-z

**Published:** 2022-07-09

**Authors:** Vijendra Singh Bhati, Ramesh Raliya

**Affiliations:** IFFCO – Nano Biotechnology Research Center, Gandhinagar, 382423 Gujarat India

**Keywords:** Analytical chemistry, Environmental chemistry, Green chemistry, Nanoscale materials

## Abstract

In this paper, a comparative analysis between the hand-held refractometer and other methods (TKN and CHNS) was accomplished for the estimation of nitrogen percentage (N%) in urea, nano urea fertilizer, and diesel exhaust fluid (DEF) solution. In order to compare the performance of all methods/devices, the detection of N% in different concentrations of urea, nano urea, and DEF were evaluated in terms of their linearity. The most important finding of this study was that the refractometer-based device revealed a good linear coefficient up to 40% urea solution (R^2^ = 0.99918) among other approaches, which means the estimation of N% is more close to the theoretical value. Moreover, the refractometer has detected the urea, nano urea, and DEF samples within 3 s which were quite fast as compared to other tested methods and no requirement of any chemicals during the sample preparation and analyses. Thus, the finding of this study suggests that a hand-held urea refractometer-based portable device can be used for onsite N% determination by the fertilizer and DEF manufacturing industries and their customers due to its low cost, low power requirement, reliable estimation, rapid N% detection, and its environmental suitability.

## Introduction

The use of urea has been increased to develop derivative products such as diesel exhaust fluid (DEF) or nano urea in addition to its conventional use for fertilizer^1–4^. The main quality of urea and its derivative products is determined by its nitrogen percentage (N%). Typically nitrogen is being measured to ensure quality, rationalize its intended use or to measure the assimilation efficiency.

The Total Kjeldahl Nitrogen (TKN) method is one of the most popular method to detect the quantitative content of N% present in organic and inorganic substances in form of ammonia (NH_3_) or ammonium ions^5,6^. In this technique, the ammonia content is analyzed after digestion of urea with copper sulphate, concentrated sulphuric acid (H_2_SO_4_), and supplement to enhance the boiling point of the solution. When NH_3_ is formed, it is further distilled into a boric acid solution in the presence of alkaline. Therefore, the formations of borate anions are then titrated with hydrochloric acid. As a result, the N% content can be estimated in urea^7,8^.

Similarly, the Dumas method also known as combustion method is considered more reliable and relatively safer as this doesn’t require using corrosive chemicals and analyzing all form of nitrogen when compared to the conventional TKN method for the determination of N% content in urea^9–11^. Based on the Dumas method, an equipment having common analyzer for carbon, hydrogen, nitrogen and sulphur (CHNS) analyser that detect N% simultaneously with C, H, and S content in the substance with high accuracy. In this method, all nitrogenous compounds are converted into nitrogen di-oxide (NO_2_) through combustion at 800–1000 °C in presence of oxygen gas that reduces to N_2_ during the flow via heated copper (Cu) and tungsten oxide (WO_3_). This step eliminates the oxygen and sulphur and while, carbon di-oxide (CO_2_) and water (H_2_O) can be removed during their interaction with carbosorb and Magnesium perchlorate (Mg(ClO_4_)_2_). Therefore, nitrogen is then measured by using a thermal conductivity detector. Additionally, CHNS detects all types of nitrogen content in urea which is normally not detected by TKN^12^.

For example, Etheridge et al. investigated the nitrogen analysis for soil, plant products, and animal nutrition lab samples by using combustion and Kjeldahl procedure^7^. After analyzing all samples, they concluded that the Kjeldahl method showed slightly lower values for N as compared to the combustion method. Similarly, Marco et al. analyzed the total nitrogen available in animal feed by adopting the Kjeldahl and combustion method^9^. In their analysis, they showed almost similar results achieved by both methods in terms of repeatability and reproducibility test. Moreover, they concluded that the combustion method was suitable for the protein analysis in animal feed as compared to Kjeldahl methods based on the cost, analysis time, and suitability with the environment.

Although, the process of N% estimation from TKN and Dumas method is reliable, however this require about 3–4 h to obtain the results, furthermore, this require handling of hazardous chemicals, high temperature, expensive equipment set-up, off-line/offsite type of analyses testing and generate waste. Therefore, TKN and CHNS feasible only with the skilled personal and well equipped laboratory who can afford the equipment-set up cost but also can properly dispose the waste generated.

Recently, urea detection based on refractometer is become popular in the fertilizer industries due to its easy operation, fast response, accurate measurement, and no use of chemicals during its operation^13^.

To the best of author’s knowledge through reported literature, there are no such studies based on the N% detection in urea and nano urea, a nanotechnology based product used for nitrogen fertilization to the crop via three different methods and their correlations. In this report, we investigate the different techniques to detect the N% content present in the conventional urea and nano urea. Primarily, we discuss the three types of techniques such as Total Kjeldahl Nitrogen (TKN), CHNS, and handheld refractometer were used for the N% detection followed by their comparison. Afterward, we correlate theoretical and experimental values of urea and nano urea estimated by different techniques. Finally, we conclude our results based on the linearity by curve fitting and other detection parameters.

## Experimental

### Apparatus

A Kjeldahl Digester (Kjeldatherm, Gerhardt Analytical System) capable of heating 8 tubes of 250 ml up to 370–395 °C and a Kjeldahl Distillation–titration Unit, (Vapodest 500, Gerhardt Analytical System) were used.

FlashSmart elemental analyzer (EA) from Thermo Fisher Scientific was utilized for the combustion, separation and detection purpose. The equipment was attached with an autosampler, a reduction oven which contains copper oxide, soda lime, and magnesium perchlorate (anhydrone), a combustion oven (containing the oxidation catalyst), a gas chromatography (GC) packed column with active carbon and a thermal conductivity detector (TCD).

A digital hand-held “Pocket” urea water refractometer (ATAGO) was used for the measurement of all samples (urea and nano urea). All measurements were performed at ambient temperature. The measurement range, ambient temperature range, and automatic temperature compensation of the refractometer is 0.0 to 55.0% Brix, 10.0 to 100 °C, and 10 to 40 °C, respectively. Additionally, the accuracy, and resolution of the refractometer is ± 0.2% Brix/ ± 1 °C, and 0.1% Brix/0.1 °C, respectively.

### Chemicals and standards

The chemicals required for TKN analyses such as potassium sulfate, sulfuric acid (95%) and boric acid were obtained from Central Drug House Ltd (CDH), India; copper sulfate pentahydrated, hydrochloric acid, and sodium hydroxide pellets from was received from Merck, India.

The gases and apparatus required for CHNS analyzer, helium (He) (99.999%), O_2_ (99.999%) were obtained from Ultra-pure gases (Gujarat, India); tin container (8 mm height, 5 mm diameter, and 19 mg weight), quartz wool, quartz reactor, electrolytic copper, all of them from Thermo Fisher Scientific Inc, (USA).

Ammonium sulfate as standard from Sigma Aldrich was used for Kjeldahl method. Sulphanilamide standard (16.30% N) for elemental analysis was provided by Thermo Fisher Scientific. Conventional urea granular, liquid nano urea and DEF solution obtained from Indian Farmers Fertilizer Cooperative Ltd (IFFCO), India.

### Methodology

#### Sample preparation

Various amount of urea granular were taken and dissolved in deionized (DI) water to prepare 0.25, 0.5, 1.0, 2.5, 5.0, 7.5, 10, 15, 25, 32.5, and 40% of urea solution. While different concentration of liquid nano urea was dissolved in DI water to prepare 1 to 10% solution of nano urea. Moreover, ultrasonication was performed to all samples ensuring that the solution mixed properly. Additionally, a DEF solution was also obtained which present 32.5% urea and 67.5% DI water. Moreover, two replicates were used for all samples (urea, nano urea, and DEF) during the analysis by different techniques.

#### Kjeldahl method (TKN)

A known amount of samples (1.0 g) was placed into 50 ml of volumetric flask and thereafter, the solution was making up to 50 ml with DI water. Then 5 ml of solution was taken from prepared solution and transferred into Kjeldahl digestion tube. After that, 2 g of catalyst (1 g of copper sulphate mixed with 10 g of potassium sulphate) and 20 ml concentrated sulfuric acid were added into Kjeldahl tube and thoroughly mixed. Meanwhile, blank samples was also prepared and placed along with all samples on digestion block followed by heating at 390 °C for 2 h. The final solution was cooled to ambient temperature and further diluted by adding 10 ml of DI water. Thereafter, all Kjeldahl tubes was placed one-by-one in the distillation-titration unit. After that, a known amount of sodium hydroxide (32%) solution was automatically added and the solution was further distilled up to 6 min. Consequently, the ammonia was collected in the 2% boric acid solution automatically and titrated against the standardized hydrochloric acid (0.1 M) until end point detection. In order to check the content of titrating solution, ammonium sulfate was used as a standard.

#### Dumas—combustion method (CHNS)

To perform the N% analyses by CHNS, 2 mg of conventional urea and nano urea samples with their different concentration were loaded into a tin container and then introduced into the combustion reactor via autosampler. A continuous helium gas flow (140 ml/min) entered in the chamber. An oxygen flow (250 ml/min) for 5 s was taken during the analysis cycle. Moreover, the temperature of the combustion reactor was maintained at 950 °C throughout the analysis. The quartz combustion reactor tube was filled from bottom to top as follows; 20 mm of quartz wool, 140 mm of electrolytic copper, 20 mm of quartz wool, 50 mm of copper oxide, and 10 mm of quartz wool. After combustion, the evolved gases were transported by a He flow via a reduction reactor filled with copper. Thereafter, the evolved gases were passed through the CO_2_, water traps, and then reached GC column and detected by TCD. The total analysis cycle was completed within 10 min. Sulphanilamide was used as a standard for the calibration of the instrument.

#### Refractometer method

A digital hand-held “Pocket” refractometer was also used to measure the different concentrations of urea (0.25 to 40%) and nano urea (1 to 10%) samples. Initially, the device was calibrated with DI water indicated zero value on the screen before use. Afterward, a few droplets of different concentrations of urea/nano urea liquid solution were placed on the prism surface of the refractometer. Therefore, the urea% of any samples can be viewed on the display of the refractometer within 3 s. Hence, the N% measurement was estimated by multiplying of 0.46 to the results acquired by the refractometer in the form of urea%.

### Statistical analyses

Statistical analyses were executed using general linear model of the OriginPro 8.5 (Origin Lab Corporation). Linear regression analyses were performed to evaluate the correlation of N% in different samples by using different analytical techniques (for example, TKN, CHNS, and refractometer).

## Results and discussion

The utilization of handheld refractometer based detectors has gained significant attention because of their small size, low-cost, portable, no need for any skilled person, and real-time detection. In this device, a few drops of liquid suspension containing urea can be placed on the prism, and therefore, the reading is displayed on the screen within 3 s. This handheld instrument works on the principal of refractive index. When a liquid sample is placed on the prism surface then the light is transmitted via the solution, while some of the light is reflected and detected by photodiodes. As a consequence, a shadow line is created whose position is closely related to the refractive index of the solution. Therefore, the refractive index or another unit of measure as per refractive index is correlated by internal software after the determination of the position of the shadow lines by using the instrument. Thus, the final concentration of liquid can be viewed on the display of the refractometer. This device is easy to calibrate by simply mounting a few drops of liquid standard or distilled water on the surface of the prism.

In order to determine whether refractometer as a substitute method to TKN and CHNS for N% estimation for conventional urea as well as nano urea fertilizers, all three techniques were systemically compared in terms of linearity, and on the basis of other performance such as cost, sample throughput, environmental acceptance, and automation capabilities. The calculation of N% in different concentrations of urea and nano urea was analyzed by TKN, CHNS, and refractometer and then compared. Table 1 shows the descriptive statistics of data utilized to compare the N% analysis by using different methods (where duplicate was taken for each samples; n = 2).

**Table 1:** Descriptive statistics of data utilized to compare TKN, CHNS, and Refractometer based methods for nitrogen analysis (N%).

Sample	Method	Theoretical (N%)	Mean (N%)	Standard deviation (SD)	CV (%)
Urea: 0.25%	TKN	0.11	− 0.10	0.04	− 0.40
	CHNS		0.02	0.00	0.00
	Refractometer		0.09	0.00	0.00
Urea: 0.5%	TKN	0.23	0.00	0.00	0.00
	CHNS		0.19	0.02	10.53
	Refractometer		0.23	0.00	0.00
Urea: 1%	TKN	0.46	0.18	0.01	5.55
	CHNS		0.37	0.00	0.00
	Refractometer		0.46	0.00	0.00
Urea: 2.5%	TKN	1.15	1.49	0.06	4.03
	CHNS		1.09	0.07	6.42
	Refractometer		1.12	0.03	2.68
Urea: 5%	TKN	2.3	2.48	0.03	1.21
	CHNS		2.3	0.06	2.61
	Refractometer		2.35	0.00	0.00
Urea: 7.5%	TKN	3.45	3.68	0.00	0.00
	CHNS		3.47	0.03	0.86
	Refractometer		3.54	0.00	0.00
Urea: 10%	TKN	4.6	4.8	0.04	0.83
	CHNS		4.6	0.04	0.87
	Refractometer		4.60	0.00	0.00
Urea: 15%	TKN	6.9	6.8	0.01	0.15
	CHNS		6.83	0.00	0.00
	Refractometer		6.78	0.00	0.00
Urea: 25%	TKN	11.5	11.86	0.08	0.67
	CHNS		11.44	0.00	0.00
	Refractometer		11.04	0.00	0.00
Urea: 32.5% (DEF)	TKN	14.95	15.27	0.19	1.24
	CHNS		14.01	2.23	15.92
	Refractometer		14.72	0.10	0.00
Urea: 40%	TKN	18.4	17.89	0.26	1.45
	CHNS		17.61	0.05	0.28
	Refractometer		17.41	0.03	0.17
Nano urea: 1%	TKN	0.46	0.48	0.07	14.58
	CHNS		0.45	0.07	15.55
	Refractometer		0.41	0.00	0.00
Nano urea: 2%	TKN	0.92	1.02	0.25	24.51
	CHNS		0.88	0.01	1.14
	Refractometer		0.87	0.00	0.00
Nano urea: 3%	TKN	1.38	1.47	0.15	10.20
	CHNS		1.30	0.00	0.00
	Refractometer		1.33	0.00	0.00
Nano urea: 4%	TKN	1.84	2.11	0.49	23.22
	CHNS		1.72	0.00	0.00
	Refractometer		1.84	0.00	0.00
Nano urea: 5%	TKN	2.3	1.85	0.04	2.16
	CHNS		2.17	0.02	0.92
	Refractometer		2.35	0.00	0.00
Nano urea: 6%	TKN	2.76	2.36	0.01	0.42
	CHNS		2.57	0.03	1.17
	Refractometer		2.76	0.00	0.00
Nano urea: 7%	TKN	3.22	2.72	0.06	2.20
	CHNS		3.01	0.06	1.99
	Refractometer		3.24	0.00	0.00
Nano urea: 8%	TKN	3.68	3.29	0.04	1.21
	CHNS		3.37	0.00	0.00
	Refractometer		3.65	0.00	0.00
Nano urea: 9%	TKN	4.14	3.57	0.04	1.12
	CHNS		3.99	0.07	1.75
	Refractometer		4.19	0.00	0.00
Nano urea: 10%	TKN	4.6	4.05	0.20	4.94
	CHNS		4.28	0.15	3.50
	Refractometer		4.62	0.03	0.65

**Figure 1:**
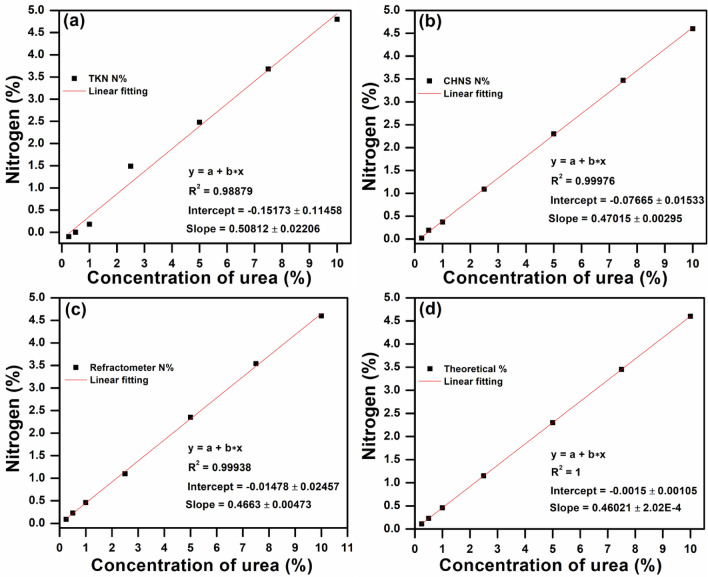
Linear fitting between nitrogen (%) and different concentration of urea from 0.25 to 10% for (**a**) TKN, (**b**) CHNS (**c**) refractometer, and (**d**) theoretical N%. (Both CHNS and refractometer have shown the R^2^ value is close to the theoretical value for urea analysis).

Moreover, their comparison has been evaluated in terms of correlations of the results obtained by a different technique. Figure 1a–d shows the linear fitting between the estimated nitrogen (%) and various concentrations of urea ranging from 0.25 to 10% for TKN, CHNS, refractometer, and theoretical value of expected N%.

**Figure 2:**
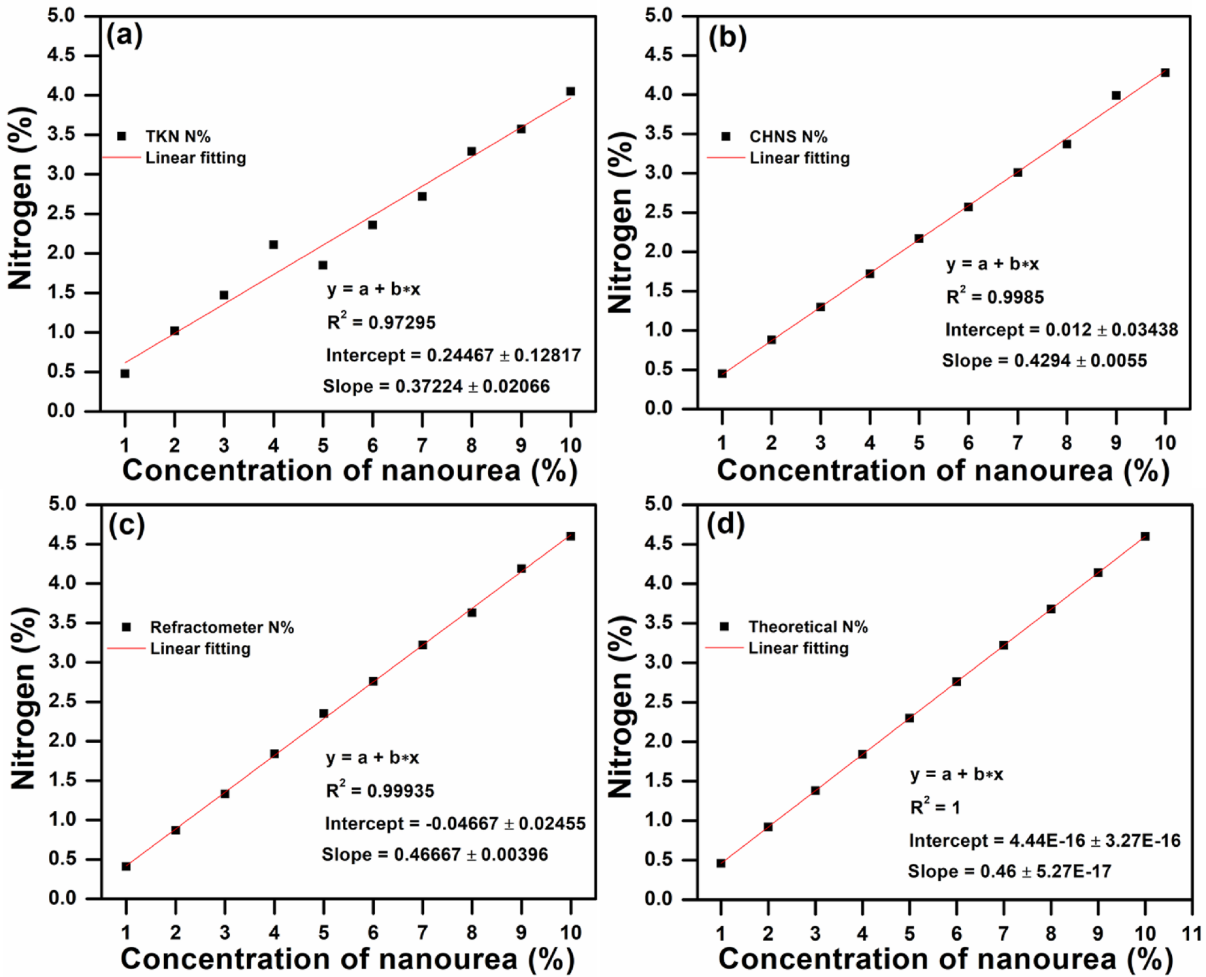
Linear fitting between nitrogen (%) and different concentration of nano urea (1–10%) for (**a**) TKN, (**b**) CHNS (**c**) refractometer, and (**d**) theoretical N%. (Refractometer showed R^2^ value is close to the theoretical value for the analysis of nano urea).

Interestingly, the refractometer was capable of detecting the lower limit of urea at 0.25% (0.11% N) and displayed 0.092% N. Moreover, it was observed that the N% detected by TKN exhibited the straight line with the lowest R^2^ (0.98879) value as compared to other techniques. Whereas, both CHNS and refractometer have detected the different concentrations of urea as their R^2^ value is close to the theoretical value of N%. Furthermore, the estimation of N% in nano urea was performed by using all techniques to evaluate the instrument performance in terms of linearity. A linear fitting between the measured N (%) and different concentrations of nano urea ranging from 1 to 10% for TKN, CHNS, and refractometer can be seen in Fig. 2a–d.

**Figure 3:**
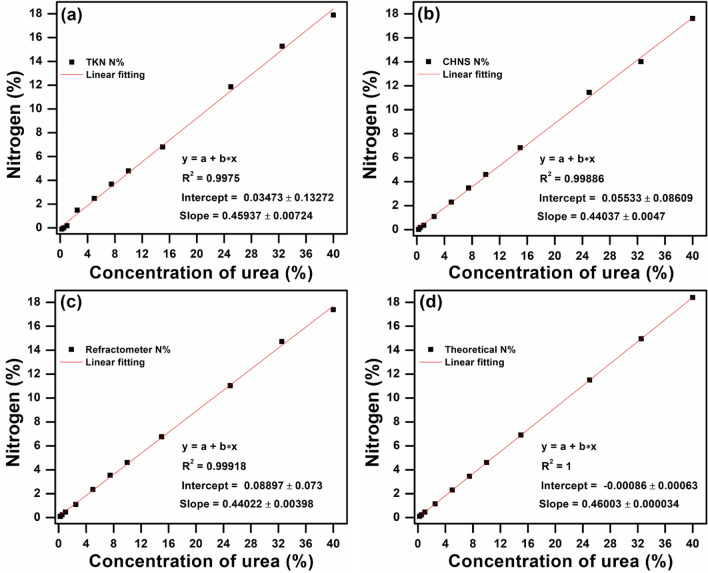
Linear fitting between nitrogen (%) and different concentration of urea ranging from 0.25 to 40% (including DEF at 32.5% urea) for (**a**) TKN, (**b**) CHNS (**c**) refractometer, and (**d**) theoretical N% (Refractometer showed R^2^ value is close to the theoretical value for urea analysis).

These results indicated that the refractometer-based device showed R^2^ = 0.99935 with an intercept of − 0.04667 ± 0.02455 and a slope of 0.46667 ± 0.00396. It can be concluded that the linear fitting for the estimation of N% in nano urea by refractometer is more closed to the theoretical value of N% as compared to other methods. Furthermore, in view of the importance of the DEF solution in diesel engines for air pollution prevention, higher concentrations of urea up to 40% were utilized for the analysis. A linear fitting between nitrogen (%) and different concentration of urea ranging from 0.25 to 40% for TKN, CHNS, refractometer, and the theoretical N% have been displayed in Fig. 3a–d. On these concentrations, the refractometer-based device produced outstanding results in terms of R^2^ (0.99918) in comparison with other techniques, which indicates that the refractometer can also be used for the analysis of DEF solution.

Table 2 displays the different techniques for the measurement of urea and nano urea sample containing concentration up to 10% and their respective values such as R^2^, intercept and slope extracted via linear fitting.

**Table 2:** Different analysis methods for various concentrations (up to 10%) of urea and nano urea and their R^2^, intercept, and slope values extracted by linear fitting.

Tech.	R^2^			Intercept			Slope		
	Urea (0.25–10%)	Nano urea (1–10%)	UREA (0.25–40%, including DEF)	Urea (0.25–10%)	Nano urea (1–10%)	Urea (0.25–40%, including DEF)	Urea (0.25–10%)	Nano urea (1–10%)	Urea (0.25–40%, including DEF)
TKN	0.98879	0.97295	0.9975	− 0.15173 ± 0.11458	0.24467 ± 0.12817	0.03473 ± 0.13272	0.50812 ± 0.02206	0.37224 ± 0.02066	0.45937 ± 0.00724
CHNS	0.99976	0.9985	0.99886	− 0.07665 ± 0.01533	0.012 ± 0.03438	0.05533 ± 0.08609	0.47015 ± 0.00295	0.4294 ± 0.0055	0.44037 ± 0.0047
Refracto-meter	0.99938	0.99935	0.99918	− 0.01478 ± 0.02457	− 0.04667 ± 0.02455	0.08897 ± 0.073	0.4663 ± 0.00473	0.46667 ± 0.00396	0.44022 ± 0.00398
Theoretical value	1	1	1	− 0.0015 ± 0.00105	4.44E − 16 ± 3.27E−16	− 0.00086 ± 0.00063	0.46021 ± 2.02E−4	0.46 ± 5.27E−17	0.46003 ± 0.000034

**Figure 4:**
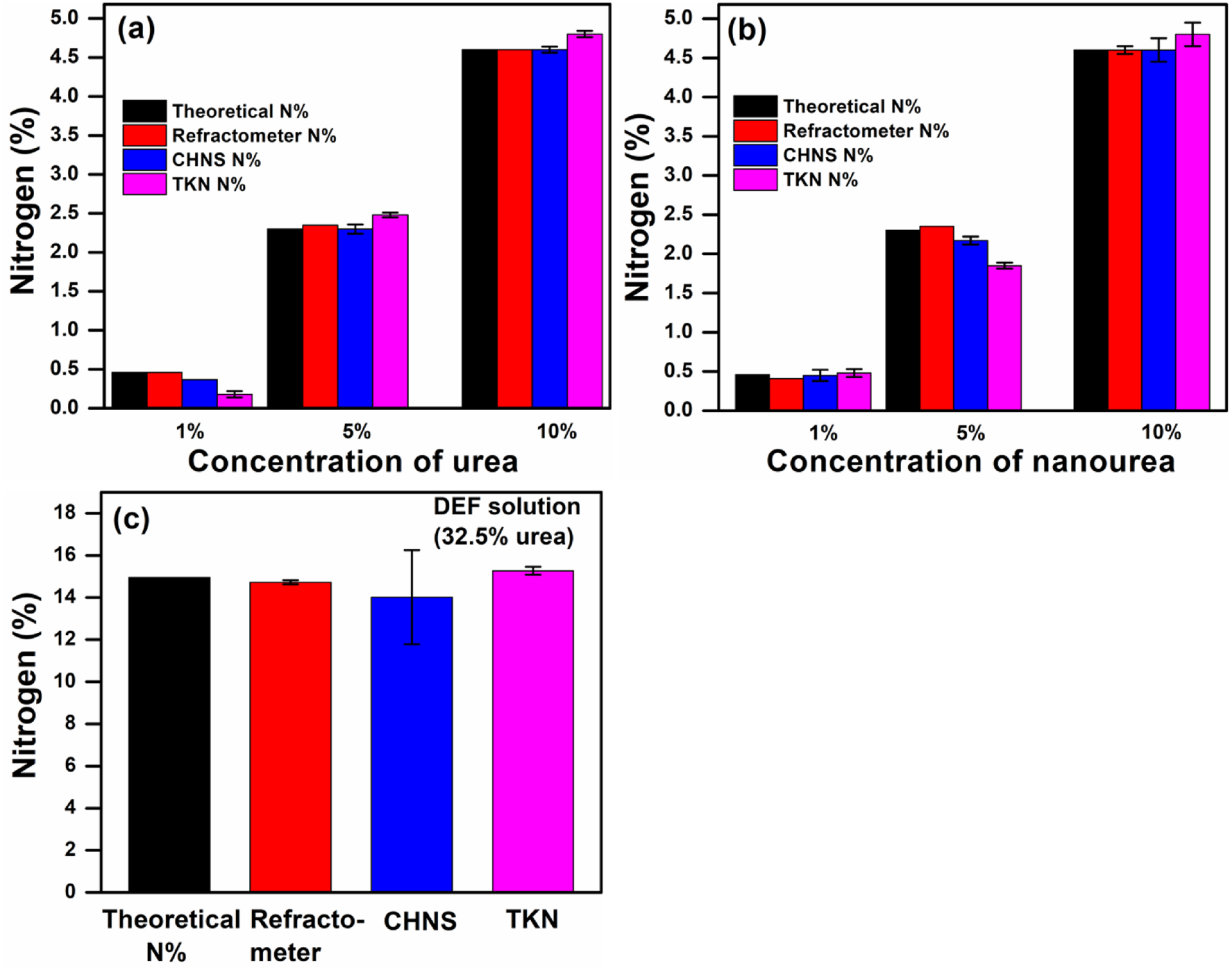
N% detection by using different techniques and their comparison with the theoretical value of different concentration of (**a**) urea and, (**b**) nano urea (**c**) estimation of N% in DEF solution containing 32.5% urea by using different methods (refractometer, CHNS, and TKN) and compared with theoretical value of N%. (Refractometer based device showed less deviation ~ 1.53% with theoretical value as compared to other techniques in case of DEF solution).

In order to further evaluate, the measured N% of different concentrations of urea and nano urea (1, 5, and 10%) were extracted by all methods and compared as shown in Fig. 4a, b.

It was observed that N% estimation by TKN is inconsistent with the theoretical value of N%. The reason is this technique only detects nitrogen from ammonium as well as organic constituents such as amino acids, nucleic acids, and proteins in the sample. However, it is not possible for the measurement of other forms of nitrogen present in nitrite and nitrate using TKN technique^8^. Moreover, refractometer-based results are more closed to that of the theoretical value of N% as compared to other techniques as shown in Fig. 4. Figure 4c displays the analysis of N% in DEF solution by utilizing different methods such as CHNS, TKN, and refractometer and then compared their results with the theoretical value of N%. In this analysis, TKN, CHNS, and refractometer based device showed 15.27, 14.01, and 14.72% N-content in DEF, respectively. It was observed that the N% was deviated about + 6.29% in TKN, − 2,14% in CHNS, and − 1.53% in refractometer with the theoretical value of N% in DEF. Therefore, the refractometer based device exhibited the more close value to theoretical N% in case of DEF.

### Other performances

Measurement time and automation: When analyzing a large number of samples in laboratories, then these characteristics have a significant concern. For example, TKN can analyze only 8 samples (including two replicates of each sample and two blank) using one digestion block for 8 tubes and one distillation-titration unit within 4 h. In the case of CHNS, around 13–17 analyses can be executed in the same period. In contrast, a refractometer can analyze about 170–180 samples (including sampling, testing, and washing the prism surface) in 4 h. Thus, this device enables the fast detection of N% for urea samples.

Dealing with the automation capabilities, TKN has some manual steps (for example, insertion of reagents in digestion tube, dilution of chemicals after digestion and positioning the digestion tube in distillation system). Moreover, samples analysis by CHNS has some advancement in terms of samples insertion via autosampler. On the other hand, one can easily monitor the results by pressing the button on a refractometer after placing a few drops of liquid samples.

Environmental and safety perspective: The utilization of hazardous acids (sulfuric acid, sodium hydroxide) and catalyst-based heavy metals are a great concern while using the TKN. Moreover, there are a small number of uses of heavy metals during the sample analysis by CHNS. On the contrary, refractometer does not require any hazardous chemicals and toxic elements during the sample analysis. Surprisingly, DI water is only used for the washing of the prism surface after the measurement.

Cost: Both instruments (TKN and CHNS) are expensive. The price of sample analysis includes their fixed (instrument cost), variable (glassware, standard chemicals, other chemicals, power, water consumption, and maintenance cost), and labor cost of a technician. On the other hand, a hand-held refractometer is quite cheap as compared to both instruments. The device operation is simple and no other cost is involved. For all these reasons, hand-held refractometer-based urea detection could be a possible technique in the field of fertilizer industries.

## Conclusions

In conclusion, a hand-held refractometer and other analytical instruments (TKN, and CHNS) were used to detect the N% in urea and nano urea fertilizer and their comparative study has been carried out. The linear correlation has been evaluated for all samples after extracting the results by using all three techniques. Among all the investigated techniques, the hand-held refractometer-based device showed good linearity (R^2^ = 0.99935) and remarked almost closed to the theoretical value in the case of nano urea. Moreover, the hand-held refractometer showed the more accurate response towards the DEF solution as compared to other techniques. Additionally, other performance parameters were also compared in terms of their cost, detection time, environmental safety issues, and portability. Such excellent features like quick detection (3 s), low power, and without the use of additional chemicals in hand-held refractometer-based device may replace the TKN and CHNS instruments for routine analysis of urea and nano urea in fertilizer industries and DEF production unit.

## Supplementary Information

Supplementary Figures.

Supplementary Figures.

## Data Availability

The datasets generated during and/or analyzed during the current study are available from the corresponding author on reasonable request. All data generated or analyzed during this study are included in this published article (and its Supplementary Information files).
