# Author Correction: Comparative estimation of nitrogen in urea and its derivative products using TKN, CHNS and hand-held refractometer

**DOI:** 10.1038/s41598-022-16797-w

**Published:** 2022-07-19

**Authors:** Vijendra Singh Bhati, Ramesh Raliya

**Affiliations:** IFFCO – Nano Biotechnology Research Center, Gandhinagar, Gujarat 382423 India

Correction to: *Scientific Reports* 10.1038/s41598-022-15736-z, published online 09 July 2022

The original version of this Article contained an error in authors Affiliation, which was incorrectly given as ‘Nano Biotechnology Research Centre, Indian Farmers Fertilizer Cooperative Limited, Gandhinagar, 382423, India’.

The correct affiliation is listed below.

IFFCO – Nano Biotechnology Research Center, Gandhinagar, Gujarat, 382423, India.

The original Article has been corrected.

