# Supplementary material for: Comparative estimation of nitrogen in urea and its derivative products using TKN, CHNS and hand-held refractometer
Source: Sci Rep. 2022 Jul 9;12:11704. doi: 10.1038/s41598-022-15736-z (PMC9271061; doi:10.1038/s41598-022-15736-z)

**Supporting information**

**Estimation of N% in conventional urea, nano urea and DEF solution: a comparative study between TKN, CHNS, and hand-held refractometer**

Vijendra Singh Bhati^a^, Ramesh Raliya^a^

*^a^Nano Biotechnology Research Centre,*

*Indian Farmers Fertilizers Cooperative Limited, Gandhinagar,-382423 India*

**Corresponding Author:**

^*^E-mail: [rameshraliya@iffco.in](mailto:rameshraliya@iffco.in), vjndrbht821@gmail.com

Phone: +91-79-23282020

**S:1** A digital photograph of digital hand-held “Pocket” refractometer for urea detection


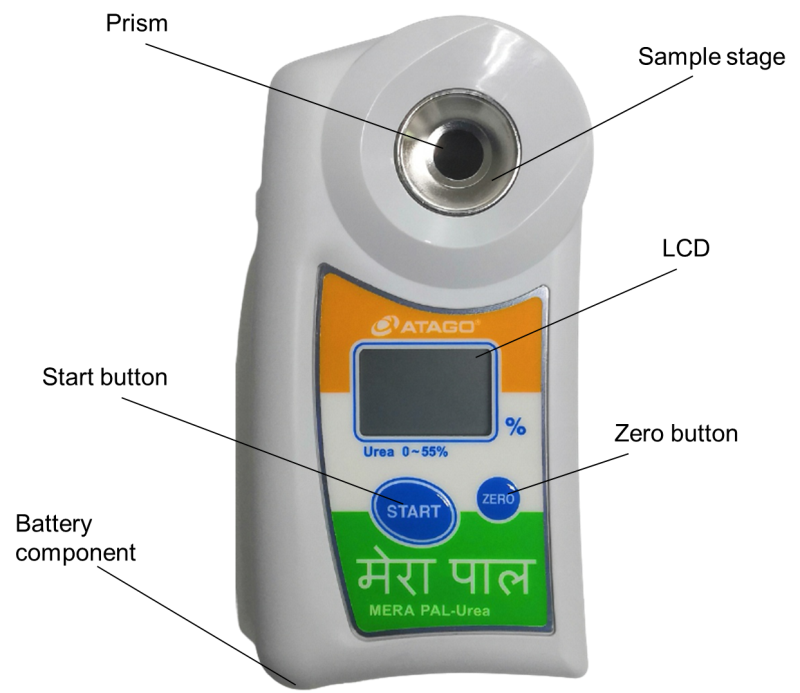


**Calculation of nitrogen (in %):** detected urea × 0.46

**Note:** 0.46 is multiplied with detected urea due to the presence of total nitrogen in urea is 46%.

**S: 2** Schematic representation of the CHNS instrument

**
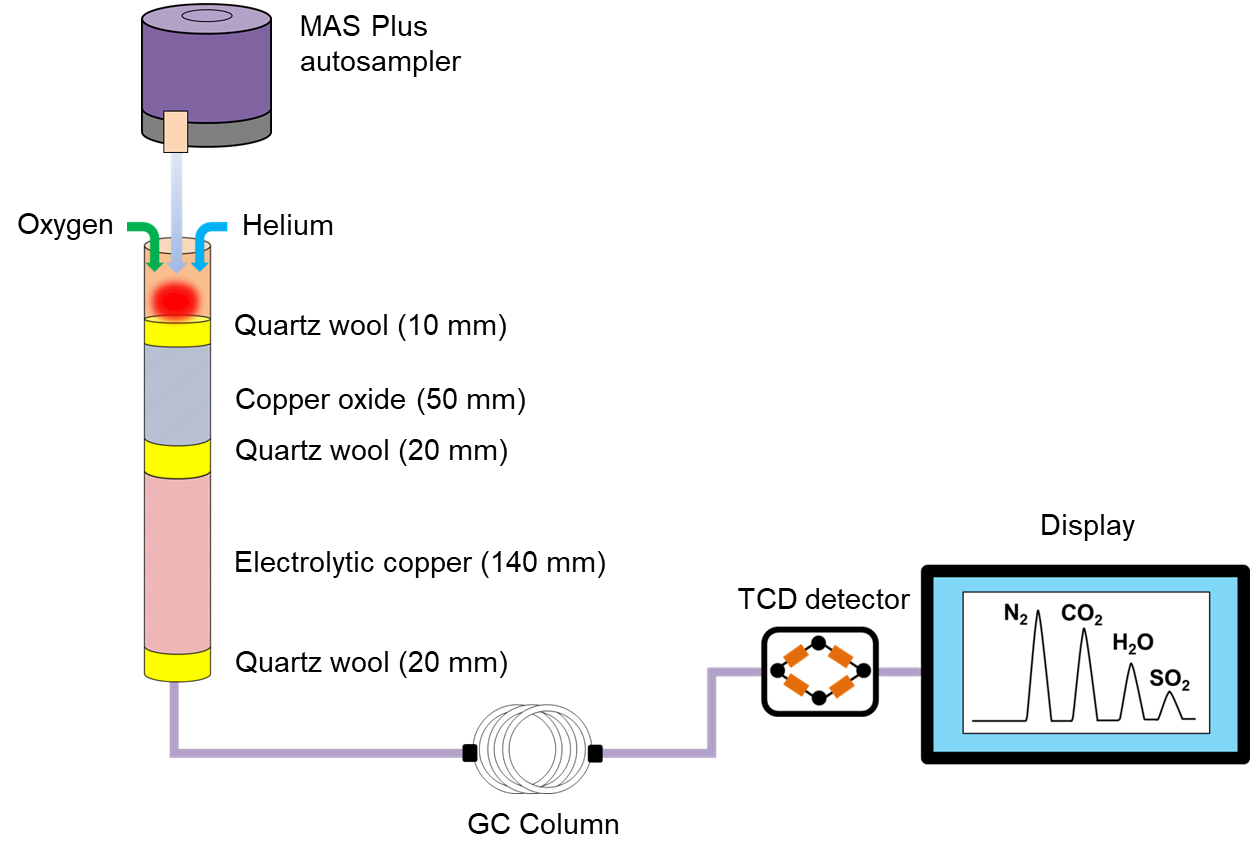
**

**S: 3** Digital image of Total Kjeldahl Nitrogen (TKN) analyzer, Gerhardt Analytical System (a) digestion unit (KJELDATHERM) (b) distillation-titration unit (VAPODEST 500)


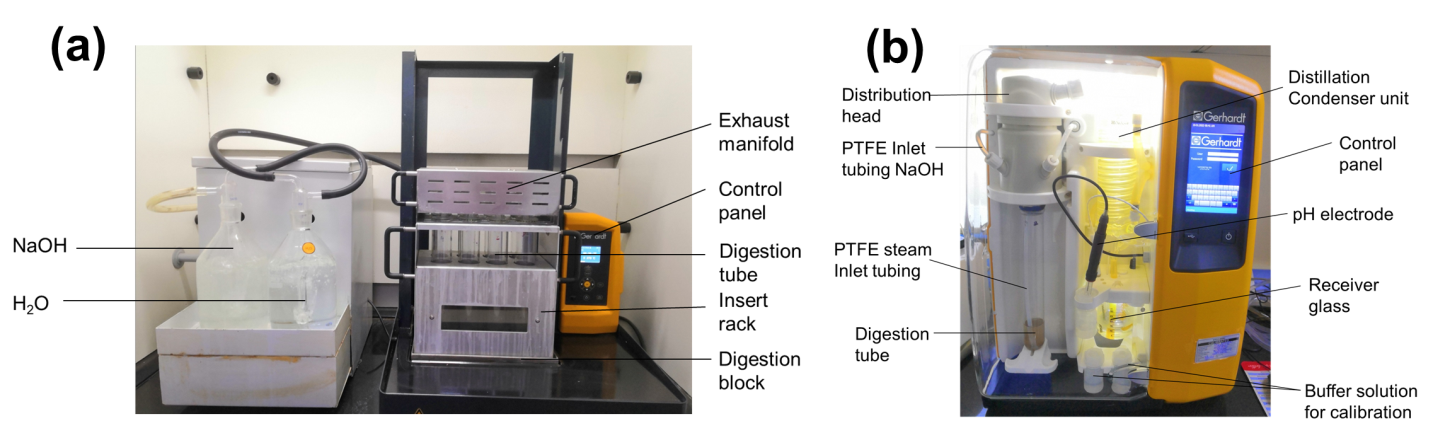

Supplement: Supplementary file 2 — Supplementary Figures. [file 41598_2022_15736_MOESM2_ESM.docx]
